# Correspondence

**Published:** 1876-01

**Authors:** 

**Affiliations:** Chicago


### Correspondence.

CHICAGO, NOV. 15, 1875.

EDITOR CHICAGO MEDICAL JOURNAL AND EXAMINER—

DEAR SIR:—An editorial appeared in the last issue of the *American Medical Weekly*, which will call forth a protest from many of the medical profession, who desire progress rather than retrogression in medical affairs. The editorial bears the title "Physicians compounding their own prescriptions," and opens by declaring "this is a subject worthy of the most careful consideration of every reader."

The writer admits the truth of *this* statement, if there is any probability of the editor's views being adopted by the profession at large, but with this exception the editor bases his argument upon erroneous statements throughout; he assumes that the apothecary is of no value to the physician, further than as a compounder of prescriptions, also that the physician is equally competent to compound his own prescriptions. In this hypothesis he ignores the fact that the practice of pharmacy involves a peculiar knowledge of Materia Medica, quite unlike that required by the physician, as also, the skill necessary to prepare from crude materials the many chemical and galenical preparations now in common use, of which the physician has only a general knowledge, enabling him to properly prescribe them in the treatment of diseases. Without this previous preparation the physician would at once find himself unable to select from the numerous useful and elegant remedies now at his disposal.

The editor also assumes, that whatever pertains to medicine is—by some sort of proprietorship—the exclusive right of the physician to make and unmake. Modesty and justice would indicate the propriety of recognizing the status of so ancient and honorable an art as that of the apothecary, which has been correctly termed "the handmaid of medicine."

If the relative merits of the two professions were to be accurately measured by the rule of medical progress, we are in doubt which would be found most worthy. Certain it is, the apothecary would have been sadly missed, by intelligent physicians, during the last hundred years, in the production of what became necessities for the modern practice of medicine.

The editor, while ignorantly admitting the services rendered by skillful apothecaries, in having made it possible for a physician to carry sufficient medicine in his pocket-case to supply each patient, coolly turns upon his benefactors with the statement that their services are no longer required. He says: "If medicines were administered in crude form, as of yore, it would be simply a matter of necessity that physicians, unable to carry with them a proper supply of medicine, should send their prescriptions to drug-stores." Who but the now useless apothecary caused this marked improvement? Has improvement in medicinal agents ceased to be desirable?

A leading argument used for dispensing medicines at the bedside, is, that each physician is losing annually from ten to fifteen hundred dollars as the profits to druggists. We fail to see how a man can lose what he never possessed, and we also fail to see the justice of appropriating the well earned fruits of labor belonging to the apothecary. If cash receipts (whether entitled to them or not) are to be the only gauge, we might suggest that the undertaker's fees are also within easy reach and they might possibly be increased if no careful apothecary stands in the way to correct errors of omission and of commission.

We have been of the opinion that the proper understanding of diseases and their treatment, was enough for the brain-work of any one man; and we have yet to meet the physician who has mastered the requirements of his profession.

Until we do meet with this order of mental and professional attainment we shall object to dispensing with our excellent assistant, the careful and conscientious apothecary, as we think each profession requires for its proper work the talents and labor of separate and equally educated minds. Whenever prescriptions are dispensed by office students from medicines prepared by "wholesale druggists," (as the editor suggests,) we think the death-rate will alarm even the "Chicago Board of Health."

No physician having a "paying practice" would care to involve himself in so much risk and laborious anxiety, while to those who have not yet reached the goal of their ambition, we would say, study and work harder to become a good physician, and leave the work of the apothecary to those best fitted for its duties.

The editor gives a further reason for his proposed innovation, by saying that the subtle and powerful influence of the mind of the physician over that of his patient is destroyed by the intervention of the apothecary. This carries us rather too far into the science of psychology, and treads too closely upon the heels of Homœopathy. We are not disposed to follow him into a discussion of the comparative influence of medicine and imagination in relieving gastritis, but would suggest that an intelligent physician should use both mind and matter, in his treatment of the sick, with such masterly skill, that the dreadful apothecary shall not overthrow him.

Lastly, we should not forget, there are two sides to all questions, and if physicians should generally dispense their own medicines we fully expect to see apothecaries—as of old—become practitioners. They are, doubtless, quite as well fitted to exert a *subtle influence* and to prescribe for a large number of patients, as the physician to dispense his own prescriptions.

Let us be just to each other, and the two professions, nobly devoted to healing the sick, may each aid the other in its beneficent work, and thereby secure the best results for mankind.

Yours in behalf of MEDICINE.

